# Children of parents with a mental illness – stigma questionnaire: validation and revision

**DOI:** 10.3389/fpsyt.2024.1376627

**Published:** 2024-06-17

**Authors:** Markus Stracke, Lisa-Marie Dobener, Hanna Christiansen

**Affiliations:** ^1^ Department of Clinical Child and Adolescent Psychology, Philipps University Marburg, Marburg, Germany; ^2^ German Center for Mental Health, Philipps University Marburg, Marburg, Germany

**Keywords:** children of parents with a mental illness, stigma, stigma by association, family stigma, questionnaire

## Abstract

**Introduction:**

Mental disorders are often stigmatized in society. The stigma of mental illness affects people with a mental illness themselves as well as their family members—a phenomenon called stigma by association (SBA). Children of parents with a mental illness (COPMI) are a particular vulnerable group for SBA. In our systematic review, *experienced SBA*, *anticipated SBA*, *affiliate SBA*, and *structural discrimination* were identified as relevant stigma dimensions for children of parents with a mental illness. To assess SBA in adolescents who grow up with a parent with a mental illness, the COPMI-SQ was developed.

**Methods:**

*N* = 930 adolescents completed the study. Of those, *N* = 380 adolescents (sample 1; 72.6% female, mean age 17.12 (*SD* = 2.01) years) reported growing up with at least one parent with a mental illness. Using confirmatory (CFA) and exploratory factor analyses (EFA) as well as standard item and reliability analyses, we analyzed and revised the COPMI-SQ in the first sample. To validate the factorial structure of the revised COPMI-SQ, CFA was also conducted in the independent sample of the other *N* = 550 adolescents (sample 2; 80.0% female, mean age 16.36 (*SD* = 1.98) years) who reported not growing up with a parent with a mental illness. To test four measurement invariance, a multiple-group CFA was conducted in the combined sample of adolescents who reported growing up with and without a parent with a mental illness (sample 1 and sample 2).

**Results:**

CFA in sample 1 resulted in an inadequate model fit for the theoretically assumed four-factor structure (CFI = .687; RMSEA = .064 (90% CI = .062–.066); SRMR = .092; AIC = 229 155.63). Following EFA and item and reliability analyses in sample 1, the COPMI-SQ was reduced to four scales (“Experienced SBA,” “Affiliate SBA,” “Shame,” and “Anticipated SBA”) and two additional screening scales (“Healthcare” and “Social support”). To facilitate questionnaire use, only the three best items were retained in each scale, reducing the total item number to 12 plus five additional screener items. CFA in sample 2 also resulted in an inadequate model fit for the theoretically assumed four factor structure (CFI = .667; RMSEA = .065 (90% CI = .063–.066); SRMR = .101; AIC = 335 651.99). In comparison, the final version of the COPMI-SQ-r showed the best model fit (CFI = .945; RMSEA = .062 (90% CI = .052–.072); SRMR = .049; AIC = 60 008.05). In the multiple-group CFA (sample 1 and sample 2), metric invariance was established (χ^2^ (208) = 481.58, p < .001; CFI = .939; RMSEA = .053 (90% CI = .047-.059); SRMR = .056). In sample 2, internal consistency was found to be good for the total scale (α = .84) and almost acceptable to almost good for the subscales (α = .64 to.78).

**Discussion:**

The revised version of the COPMI-SQ (COPMI-SQ-r) is a reliable and economic questionnaire to assess SBA in adolescents who grow up with a parent with a mental illness. The COPMI-SQ-r can be used to help develop and evaluate anti-stigma and general interventions for affected adolescents.

## 1 Introduction

Since mental disorders are still frequently stigmatized in society, the World Health Organization (WHO) calls in its “Comprehensive Mental Health Action Plan 2013–2030” for targeted actions to lower the stigmatization of people with mental disorders (1). The term stigma (Greek for stab, burn, or wound mark) goes back to the ancient Greeks, who cut or burned marks into the skin of criminals or slaves to identify them as corrupt or immoral people to be avoided (2). Today, stigmatization is understood as a process in which individuals are assigned to a group based on a characteristic or trait that is consensually categorized by society as deviating from the norm and therefore devaluated (3, 4). Stigmata are not inherent in the individuals themselves but are social constructs and depend on historically shaped cultural norms (4). The process of stigmatization consists of cognitive (stereotypes), emotional (prejudices), and behavioral (discrimination) components (5, 6). Stereotypes are simplified and generalized knowledge structures about social groups. The approval of the existing stereotype in association with negative emotions creates a prejudice, which, in turn, can result in a behavioral reaction in the form of discrimination (5, 6). Taken together, stereotypes and prejudices in the society are also referred to as public stigma (7). Negative stereotypes of people with mental disorders include stereotypical assumptions of character weakness, incompetence, dangerousness, or unpredictability (6, 8). The stigmatization of people with mental disorders is associated with serious and far-reaching consequences for those affected. Some authors argue that the consequences of stigma are actually worse than those of the mental disorder itself (9). Experiences of discrimination due to a mental disorder can lead to less help seeking (10), social isolation, hopelessness, and suicidal thoughts from internalizing the stigma (11). For those affected by stigmatization, three mechanisms are known to be most relevant (5): *experienced stigma*, *anticipated stigma*, and *internalized stigma*. Whereas *experienced stigma* refers to personally experienced stereotypes, prejudices, and discriminations in past or present, *anticipated stigma* represents the expectation of stigmatization in the future. Last but not least, *internalized stigma*, which is also called *self-stigma* (12), is defined as the internalization of stereotypes and prejudices to the self.

Regarding various discredited groups affected by stigmatization (e.g., people with AIDS, physical or mental illness), there is evidence that both the trait-bearing individuals themselves experience and expect stigmatization as well as people associated with them (13–16). This form of stigmatization, first referred to as *courtesy stigma* (2), is nowadays called *stigma by association* (SBA) (17). SBA depends on the entitativity, that is, the degree to which the trait-bearing individual and other associated people are perceived as a social unit. Since the highest degree of entitativity is attributed to families (17, 18), family members carry a higher risk of experiencing SBA. When SBA exists within families, it is also called *family stigma* (19). In terms of the stigmatization of people with mental disorders, this means that the whole family system experiences stigmatization due to the mental illness of one family member. Embarrassment, shame, guilt, and fear of contamination are reportedly frequent consequences of *family stigma* concerning mental illness (20–22).

Worldwide, every fourth child and adolescent grows up with at least one parent with a mental illness (23, 24). Children and adolescents who grow up with a parent with a mental illness are a particularly vulnerable group to develop a mental disorder themselves (25–28). A recent large meta-analysis reported a lifetime risk of 55% among those children and adolescents to develop any mental disorder (27), whereby subclinical symptoms occur more often and earlier in life (29). Due to their close and dependent relationship to their parents, these children and adolescents are also considered to be particularly affected by SBA (21, 30) and stigmatization has been identified as a potential social mechanism in the transgenerational transmission of mental disorders (TTMD) (29).

Thus, to reduce the stigma of mental illness in general and SBA in children of parents with a mental illness specifically, we need to develop general anti-stigma campaigns and targeted interventions to reduce SBA in children and adolescents who grow up with a parent with a mental illness (1, 31). To make this possible, a better understanding of the specific stigma experiences of affected children and adolescents is necessary, as are valid questionnaires to assess this information. Analogous to the mechanisms concerning the stigmatization of people with mental disorders in general (5), our recent systematic mixed studies review identified *experienced stigma*, *anticipated stigma*, *affiliate stigma*, and *structural discrimination* as important stigma dimensions for children and adolescents who grow up with a parent with a mental illness (32). *Affiliate stigma* (33) can be understood as the SBA version of the aforementioned *internalized stigma* or *self-stigma* (5, 12, 32, 33). *Structural discrimination* refers to the societal and policy structures perpetuating stigmatization (34). Since—to our knowledge—no adequate scale measuring SBA in adolescents who grow up with a parent with a mental illness exists, we developed the Children of Parents With a Mental Illness-Stigma Questionnaire (COPMI-SQ) (30) based on the identified stigma dimensions that are specifically relevant for children and adolescents who grow up with a parent with a mental illness (32). In our first pilot study (*N* = 32), we observed the COPMI-SQ’s promising psychometric properties, but as our sample was too small, analysis of the factor structure of the COPMI-SQ was not possible.

Therefore, the present study aims to report on the factor structure of the COPMI-SQ and its validation in a larger sample. A valid instrument to measure SBA in adolescents who grow up with a parent with a mental illness will enable us to better understand their stigma experiences and can be used to help develop and evaluate anti-stigma and general interventions for affected adolescents.

## 2 Methods

### 2.1 Ethics

This study was approved by the Ethics Committee of the Department of Psychology at Philipps University Marburg. Prior to study participation, adolescents were given information about the study and gave their informed consent. If they were under the age of 18, information was also given to their legal guardians, and their informed consent was also obtained. Data were collected anonymously. As long as the questionnaire was not submitted to the online platform, participants could withdraw from answering the questionnaire at any time and without giving any reason. In case of distress due to study participation, participants could contact clinically trained study personnel at any time. None of the participants took advantage of this offer.

### 2.2 Participants

Eligible participants were all German-speaking adolescents between 12 and 21 years of age who reported growing up with at least one parent with a mental illness. No further inclusion or exclusion criteria were applied. Since recruiting children of parents with a mental illness has proven to be difficult (35, 36), adolescents who reported not growing up with a parent with a mental illness could also participate in this study and a parallel version of the COPMI-SQ that only differed on item stem was presented to them.

### 2.3 Procedure

Participants were recruited Germany-wide through an ongoing research project concerning children of parents with a mental illness (23, 37), university mailing lists, and social media advertising on Instagram and also by directly contacting (social) services for children and adolescents who grow up with a parent with a mental illness. Data were collected between January and March 2023 using the online platform SoSci Survey (38) (www.soscisurvey.de). The survey was programmed so that all items had to be answered as it was otherwise automatically terminated. At the beginning of the survey, participants completed sociodemographic questions (age, sex, socioeconomic status) as well as questions regarding their parents’ and their own mental health. Socioeconomic status was assessed using the four item Family Affluence Scale and accordingly rated as low, medium, or high (39). At the end of the survey, participants were offered to take part in a prize draw as compensation for study participation.

### 2.4 COPMI-SQ

Based on a systematic review (32), the COPMI-SQ (30) was created as an instrument to assess the stigma experiences of adolescents who grow up with a parent with a mental illness in daily life. According to theory, the items were expected to load on the four different scales “Experienced SBA,” “Anticipated SBA,” “Affiliate SBA,” and “Structural Discrimination”. In a first pilot study with *N* = 32, the item number of the COPMI-SQ was reduced from initially 109 to 67. Items are rated from 1 (“does not apply at all”) to 101 (“fully applies”) on a 100-point visual Likert scale. Cronbach’s alpha for the subscales was good to excellent (α = .87 to.95), and excellent for the total scale (α = .98). Item difficulties ranged between *P_i_* = 15.00 and *P_i_* = 77.69. Corrected item-whole correlations for the subscales ranged between *r_it_* = .28 and *r_it_* = .91. For the total scale, no corrected item-whole correlations were reported. Further information is found in **Table 1**.

**Table 1:** Characteristics of original COPMI-SQ scales.

Scale	Item no.		Sample by Dobener et al., 2022			Sample 1	
		α	*r_it_* (M (SD) [min; max])	*P_i_* (M (SD) [min; max])	α	*r_it_* (M (SD) [min; max])	*P_i_* (M (SD) [min; max])
Experienced SBA	17	.95	.71 (.16) [.40;.91]	27.36 (7.36) [15.95; 46.88]	.89	.56 (.18) [.15;.75]	21.39 (13.22) [8.01; 47.50]
Anticipated SBA	16	.95	.72 (.15) [.43;.88]	33.59 (7.08) [21.22; 44.59]	.90	.60 (.14) [.27;.75]	25.78 (11.04) [11.50; 45.31]
Affiliate SBA	19	.93	.63 (.10) [.45;.82]	29.01 (7.84) [15.00; 46.88]	.91	.57 (.08) [.43;.72]	38.29 (10.42) [20.46; 56.53]
Structural discrimination	16	.87	.51 (.13) [.28;.71]	51.47 (14.91) [28.31; 77.69]	.71	.31 (.12) [.08;.51]	63.15 (13.05) [39.41; 84.30]
COPMI-SQ-total	67	.98	nr	34.71 (13.44) [15.00; 77.69]	.95	.46 (.19) [-.09;.68]	36.58 (19.62) [8.01; 84.30]

Item no., number of items; α, Cronbach’s alpha; *r_it_*, corrected item-whole correlation; *P_i_*, item difficulty. nr, not reported.

For adolescents who reported not growing up with a parent with a mental illness, a parallel version of the COPMI-SQ was created. All item stems were adapted without changing the meaning of the items: e.g., “Because my mother/my father has a mental illness, others make fun of my mother/my mother.” was reformulated to “Because the parent of a child has a mental illness, others make fun of the parent”.

### 2.5 Statistical analysis

Statistical analyses were conducted with RStudio (40) using the packages “psych” (41), “nFactors” (42), and “lavaan” (43). For all analyses, p-values ≤.05 were set as thresholds for statistical significance.

Since rules of thumb for confirmatory factor analyses (CFA) propose a ratio of number of items to number of participants (*N*) of 1 to 5 (44), a sample size of at least *N* = 335 was calculated in advance (67 items to 335 participants).

Initially, a CFA with robust maximum likelihood estimations was conducted in sample 1 to test the theoretically derived four-factor structure of the COPMI-SQ. Goodness of fit was assessed using the chi-square test statistics, the χ2/df ratio, the root mean square error of approximation (RMSEA), the standardized root mean squared residual (SRMR), the comparative fit index (CFI), and the Akaike information criterion (AIC) (45, 46). According to conventions, non-significant chi-square test statistics, RMSEA <.08, SRMR <.08, and CFI ≥.90 are regarded as good model fit (45). Concerning AIC, the model with the lowest value is regarded as the best model fit, as it is the most parsimonious one (46). This also applies to the χ2/df ratio, although values between 2 and 3 are also considered a good fit here (47).

Since an inadequate model fit was found for the theoretically derived four-factor structure, subsequent exploratory factor analyses (EFA) were conducted in sample 1 to further investigate the factor structure of the COPMI-SQ. Using Bartlett’s test (48) and Kaiser–Meyer–Olkin (KMO) criterion (49, 50), the suitability of the data for EFA was tested. EFA was conducted using maximum-likelihood factor analysis with promax rotation. Factor extraction was based on Velicer’s MAP test and Horn’s parallel analysis as well as content considerations (51). Step by step, all items with factor loadings <.3 or with factor cross loadings ≤.2 were removed and EFA repeated until a clear factorial structure was found. At all steps, Bartlett’s test and Kaiser–Meyer–Olkin criterion were used to test the suitability of the data for EFA, and factor extraction was based on MAP test, parallel analysis, and content considerations.

Subsequently, item analyses were performed in sample 1 based on item difficulties, item discriminatory power (corrected item-whole correlations), and Cronbach’s alpha if the item is deleted. Furthermore, Cronbach’s alpha was calculated as reliability coefficient of the scales and subscales. If scales consisted of fewer than three items, the Spearman–Brown coefficient *r_SB_* was used as reliability coefficient. Item and scale analyses were always first conducted for the subscales, then for the total scale. Cronbach’s alpha of ≥.7 was deemed acceptable, ≥.8 good, and ≥.9 excellent (52). The distribution of item difficulties was aimed to be 20 ≤ *P_i_* ≤ 80 (53). Corrected item-whole correlations of.3 ≤ *r_it_* ≤.5 were regarded as acceptable and.5 < *r_it_* <.07 as good discriminatory power (53). All items with item difficulties *P_i_* < 20 or *P_i_* > 80 and with corrected item-whole correlations *r_it_* <.3 were removed.

Results of the EFA were subjected to CFA in sample 2 to confirm the derived factor structure. Furthermore, a multiple-group CFA was conducted in the combined sample of adolescents who reported growing up with and without a parent with a mental illness (sample 1 and sample 2) to test for measurement invariance. Finally, item analyses as described above were also performed in sample 2 for the derived factor structure.

## 3 Results

### 3.1 Participants

Our survey was accessed 6,649 times. Among the participants, 2,512 gave informed consent, 2,312 met the inclusion criteria (language, age), and 930 completed the survey. Of these, 380 also reported growing up with at least one parent with a mental illness and were included in sample 1. The remaining 550 adolescents who reported that their parents have no a mental illness were included in sample 2.

In sample 1, participating adolescents were mostly female (72.6%) and on average 17.12 years old (*SD* = 2.01). Almost all adolescents reported a medium (45.3%) to high (47.1%) socioeconomic status. 91.3% of the participating adolescents indicated having a mental illness themselves. Of those, almost half (46.4%) reported having more than one relevant diagnosis and 59.4% stated that they were undergoing treatment for their mental illness. Adolescents’ mental disorders were mostly classified as depressive disorders (62.0%), other symptoms (34.5%), and anxiety disorders (27.4%). Concerning the parental mental illness, almost half (47.1%) of the participating adolescents reported that only their mother and 24.5% that only their father was affected. 28.4% of the adolescents reported that both their parents were affected. Most common parental disorders were depressive disorders (61.3% for mothers, 55.2% for fathers), personality disorders (13.9% for mothers, 12.4% for fathers), and other symptoms (12.9% for mothers, 14.4% for fathers). More than one relevant diagnosis was reported for 26.8% of the mothers and for 21.4% of the fathers reported to have a mental illness. Furthermore, participating adolescents indicated that 43.2% of the mothers and 30.8% of the fathers reported to have a mental illness were being treated for their mental illness. More detailed sample characteristics for sample 1 are provided in **Table 2**.

**Table 2:** Participants sample 1, *N* = 380.

Sociodemographics and mental health status					
Gender	Female	276 (72.6%)	Parental mental illness	Mother	179 (47.1%)
	Male	43 (11.3%)		Father	93 (24.5%)
	Other	55 (14.5%)		Both	108 (28.4%)
	ns	6 (1.6%)	Comorbidity mother^2^	No	210 (73.2%)
Age	M	17.12		Yes	77 (26.8%)
	SD	2.01	Treatment mother^2^	No	148 (51.6%)
	Range	12 - 21		Yes	124 (43.2%)
SES	Low	29 (7.6%)		ns	15 (5.2%)
	Medium	172 (45.3%)	Comorbidity father^3^	No	158 (78.6%)
	High	179 (47.1%)		Yes	43 (21.4%)
Mental illness	No	33 (8.7%)	Treatment father^3^	No	126 (62.7%)
	Yes	347 (91.3%)		Yes	62 (30.8%)
Comorbidity^1^	No	186 (53.6%)		ns	13 (6.5%)
	Yes	161 (46.4%)			
Treatment^1^	No	141 (40.6%)			
	Yes	206 (59.4%)			
Diagnosis^4^			Adolescents^1^	Mothers^2^	Fathers^3^
Neurodevelopmental disorders			60 (17.3%)	16 (5.6%)	18 (9.0%)
Schizophrenia and other psychotic disorders			1 (0.3%)	10 (3.5%)	9 (4.5%)
Bipolar and related disorders			4 (1.2%)	17 (5.9%)	17 (8.5%)
Depressive disorders			215 (62.0%)	176 (61.3%)	111 (55.2%)
Anxiety disorders			95 (27.4%)	33 (11.5%)	8 (4.0%)
Obsessive-compulsive and related disorders			19 (5.5%)	7 (2.4%)	0 (0.0%)
Trauma- and stressor-related disorders			54 (15.6%)	23 (8.0%)	13 (6.5%)
Dissociative disorders			12 (3.5%)	4 (1.4%)	0 (0.0%)
Feeding and eating disorders			54 (15.6%)	11 (3.8%)	1 (0.5%)
Sleep–wake disorders			5 (1.4%)	1 (0.3%)	0 (0.0%)
Gender dysphoria			1 (0.3%)	0 (0.0%)	0 (0.0%)
Substance-related and addictive disorders			1 (0.3%)	11 (3.8%)	23 (11.4%)
Personality disorders			49 (14.1%)	40 (13.9%)	25 (12.4%)
Other^5^			120 (34.5%)	37 (12.9%)	29 (14.4%)

Information is based solely on information provided by participating adolescents; ns, not stated; SES, socioeconomic status. ^1^Percentages refer to *n* = 347 adolescents who stated having a mental illness. ^2^Percentages refer to n = 287 mothers reported to have a mental illness according to the participating adolescents. ^3^Percentages refer to n = 201 fathers reported to have a mental illness according to the participating adolescents. ^4^(Parental) mental disorders were reported by adolescents in a free-text answer and clustered according to DSM-5 categories (54). ^5^For example, listing of symptoms, suspected diagnoses, or other problems.

In sample 2, participating adolescents were mostly female (80.0%) and on average 16.36 years old (*SD* = 1.98). Almost all adolescents reported a medium (35.6%) to high (62.2%) socioeconomic status. 68.4% of the participating adolescents indicated having a mental illness themselves. Of those, 35.6% reported having more than one relevant diagnosis and 39.6% stated that they were undergoing treatment for the mental illness. Adolescents’ mental disorders were mostly classified as depressive disorders (54.0%), anxiety disorders (33.0%), and other symptoms (26.6%). More detailed sample characteristics for sample 2 are provided in **Table 3**.

**Table 3:** Participants sample 2, *N* = 550.

Sociodemographics and mental health status					
Gender	Female	440 (80.0%)	Mental illness	No	174 (31.6%)
	Male	48 (8.7%)		Yes	376 (68.4%)
	Other	47 (8.5%)	Comorbidity^1^	No	242 (53.6%)
	ns	15 (2.7%)		Yes	134 (35.6%)
Age	M	16.36	Treatment^1^	No	227 (60.4%)
	SD	1.98		Yes	149 (39.6%)
	Range	12 - 21			
SES	Low	12 (2.2%)			
	Medium	196 (35.6%)			
	High	342 (62.2%)			
Diagnosis^2^				Adolescents^1^	
Neurodevelopmental disorders				35 (9.3%)	
Schizophrenia and other psychotic disorders				3 (0.8%)	
Bipolar and related disorders				6 (1.6%)	
Depressive disorders				203 (54.0%)	
Anxiety disorders				124 (33.0%)	
Obsessive-compulsive and related disorders				10 (2.7%)	
Trauma- and stressor-related disorders				27 (7.2%)	
Feeding and eating disorders				40 (10.6%)	
Sleep–wake disorders				1 (0.2%)	
Substance-related and addictive disorders				2 (0.5%)	
Personality disorders				24 (6.4%)	
Other^3^				100 (26.6%)	

Information is based solely on information provided by participating adolescents. ns, not stated; SES, socioeconomic status. ^1^Percentages refer to *n* = 376 adolescents who stated having a mental illness. ^2^Mental disorders were reported by adolescents in a free-text answer and clustered according to DSM-5 categories (54). ^3^For example, listing of symptoms, suspected diagnoses, or other problems.

### 3.2 Initial scale and item analyses (sample 1)

Initially, item and scale analyses were conducted for the theoretically derived four-factor structure in sample 1. Cronbach’s alpha was good to excellent (α >.89) for all subscales as well as the total scale (α = .95), except for the subscale “Structural Discrimination” (acceptable with α = .71). Item difficulties ranged between *P_i_* = 8.01 and *P_i_* = 84.30. Corrected item-whole correlations for the subscales ranged between *r_it_* = .08 and *r_it_* = .75, and for the total scale between *r_it_* = -.09 and *r_it_* = .68. Further information can be found in **Table 1**.

### 3.3 Confirmatory factor analysis of the original COPMI-SQ scales (sample 1)

Testing the theoretically derived four-factor structure in sample 1 with a CFA resulted in an inadequate model fit (χ^2^(2138) = 5495.92, *p* <.001; RMSEA = .064 (90% CI = .062-.066); SRMR = .092; CFI = .687; AIC = 229 155.63) (see **Table 4**).

**Table 4:** Results of confirmatory factor analysis (sample 1).

Model	χ^2^	*df*	*p*	χ^2^/*df*	RMSEA [90% CI]	SRMR	CFI	AIC
A	5 495.92	2138	<.001	2.57	.064 [.062;.066]	.092	.687	229 155.63

A, original COPMI-SQ; χ^2^, model chi square; df, degrees of freedom; *p*, p-value RMSEA, root mean square error of approximation; SRMR, standardized root mean square residual; CFI, comparative fit index; AIC, Akaike information criterion.

### 3.4 Exploratory factor analyses (sample 1)

Subsequent exploratory factor analyses were thus conducted in sample 1 to further analyze the factorial structure. According to Bartlett’s test (χ^2^(2211) = 14,038.49, *p* <.001) and Kaiser–Meyer–Olkin criterion (KMO = .92), the data were suitable for EFA. MAP test recommended the extraction of nine, parallel analysis the extraction of eight factors. Due to content considerations, eight factors accounting for 41% of the variance were initially extracted. Step by step, all items with factor loadings <.3 and with cross loadings with differences ≤.2 were deleted and EFA repeated five times. At all steps, Bartlett’s test and Kaiser–Meyer–Olkin criterion revealed the data’s suitability for EFA. For factor extraction, we considered the MAP test, parallel analysis, and content at all steps. Finally, EFA resulted in seven factors explaining 49% of the variance.

### 3.5 Item reduction 1 (sample 1)

The subscales’ items derived from the EFA and the reduced total scale were named according to their content and then analyzed and revised in sample 1 concerning their difficulty, discriminatory power, and reliability. Detailed characteristics of the reduced scales are found in **Table 5**.

**Table 5:** Results of first scale and item analyses (sample 1).

		N	M (SD) [min; max]	Item no.	α	r_it_ (M (SD) [min; max])	P_i_ (M (SD) [min; max])
Factor 1	Experienced SBA	380	24.34 (25.30) [1; 101]	3	.67	.49 (.03) [.45;.52]	23.34 (3.44) [20.40; 27.12]
Factor 2	Affiliate stigma	380	45.09 (25.27) [1; 99.55]	11	.88	.60 (.09) [.46;.76]	44.09 (6.80) [29.52; 56.53]
Factor 3	Shame	380	31.63 (24.00) [1; 97.17]	6	.80	.55 (.08) [.49;.69]	30.63 (7.22) [20.46; 39.13]
Factor 6	Anticipated SBA	380	35.01 (25.08) [1; 101]	4	.77	.57 (.07) [.48;.64]	34.01 (10.75) [22.72; 45.31]
Total***		380	37.45 (20.81) [1; 94.96]	24	.92	.54 (.09) [.3;.71]	36.45 (10.38) [20.40; 56.53]
Factor 4	Healthcare	181	72.86 (25.57) [1.33; 101]	3	.81	.66 (.02) [.64;.69]	71.86 (6.15) [64.81; 76.13]
Factor 7	Social support	380	42.86 (30.26) [1; 101]	2	.67*	.67 (.00) [.67;.67]	41.86 (3.86) [39.13; 44.59]
Total		380**	39.46 (19.39) [1; 91.92]	29	.91	.48 (.15) [.15;.69]	40.49 (.14.54) [20.4; 76.13]

Item no., number of items; α, Cronbach’s alpha; *r_it_*, corrected item-whole correlation; *P_i_*, item difficulty. *Spearman Brown coefficient *r_SB_*. **Items in factor 4 N = 181. ***Without factor 4 and factor 7.

Factor 1: All but one of the initial items in the first factor were part of the original “Experienced SBA” scale. Thus, factor 1 was named “Experienced SBA”. Initial Cronbach’s alpha was α = .91. Removing any item would not have increased Cronbach’s alpha. All items showed desirable discriminatory power .5 < *r_it_* <.7. Nine items showed item difficulties of *P_i_* < 20 and were thus removed. After removal, Cronbach’s alpha was almost acceptable (α = .67) for the remaining three items. The discriminatory power of the remaining three items was acceptable to desirable (.49 ≤ *r_it_* ≤.53) and item difficulties ranged from *P_i_* = 20.40 to *P_i_* = 27.12.

Factor 2: All items in the second factor were part of the original “Affiliate stigma” scale. Thus, factor 2 was named “Affiliate stigma”. Initial Cronbach’s alpha was α = .88. Removing any item would not have increased Cronbach’s alpha. All items showed desirable item difficulties 20 ≤ *P_i_* ≤ 80. Two items showed almost desirable discriminatory power .46 ≤ *r_it_* ≤.5. All other items showed desirable discriminatory power *r_it_* >.50. Thus, no items were removed.

Factor 3: The items in the third factor were originally part of the scales “Anticipated SBA” and “Affiliate stigma”. All items dealt with aspects of keeping the parental mental disorder a secret or being ashamed by the parental mental disorder, thus, factor 3 was named “Shame”. Cronbach’s alpha was α = .80. Removing any item would not have increased Cronbach’s alpha. All items showed desirable item difficulties 20 ≤ *P_i_* ≤ 80. One item showed almost desirable discriminatory power of *r_it_ = .*49. All other items showed desirable discriminatory power *r_it_* >.50. Thus, no items were removed.

Factor 4: All items in the fourth factor were originally part of the healthcare subscale of the “Structural discrimination” scale. Thus, factor 4 was named “Healthcare”. Cronbach’s alpha was α = .74. Removing one item improved Cronbach’s alpha to α = .81. Afterward, all three remaining items showed desirable discriminatory power *r_it_* >.5 and desirable item difficulties 20 ≤ *P_i_* ≤ 80. Thus, no further items were removed.

Factor 5: All items in the fifth factor were originally part of the “Anticipated SBA” scale. Thus, factor 5 was named “Anticipated SBA”. Initial Cronbach’s alpha was α = .87. Removing one item would have increased Cronbach’s alpha to α = .88. All items showed desirable discriminatory power *r_it_* >.5. Since all items showed inacceptable item difficulty *P_i_* <.2, all items from factor 5 were removed.

Factor 6: All but one of the initial items in the sixth factor were part of the original “Anticipated SBA” scale. Thus, factor 6 was named “Anticipated SBA”. Initial Cronbach’s alpha was α = .71. Removing one item improved Cronbach’s alpha to α = .77. Afterwards, one item showed almost desirable discriminatory power of *r_it_ = .*48. All other items showed desirable discriminatory power *r_it_* >.5. All four remaining items showed desirable item difficulties 20 ≤ *P_i_* ≤ 80. Thus, no further items were removed.

Factor 7: The items in the seventh factor were part of the original “Experienced SBA” scale and dealt with having somebody to talk to in times of need. Thus, factor 7 was named “Social support”. Since factor 7 consists of only two items, Spearman’s brown coefficient (*r_SB_*) was calculated as reliability coefficient. Spearman’s brown coefficient was acceptable with *r_SB_* = .67. All items showed desirable discriminatory power (*r_it_* >.5) and item difficulty (20 ≤ *P_i_* ≤ 80). Thus, no items were removed.

Total scale: Cronbach’s alpha was α = .91 for the total scale. Removing any item would not have improved Cronbach’s alpha. All items showed desirable item difficulties 20 ≤ *P_i_* ≤ 80. Five items showed inacceptable discriminatory power *r_it_* <.3. Since all these items loaded on factor 4 (“Healthcare”) as well as factor 7 (“Social support”) those factors were excluded from calculating the total scale. Since stigma experiences in the healthcare system (factor 4) and social support (factor 7) were considered important aspects to assess, the subscales were nevertheless retained as separate screening scales. After excluding factors 4 and 7, Cronbach’s alpha was α = .92. Removing any further item would not have improved Cronbach’s alpha. All remaining items showed desirable item difficulties 20 ≤ *P_i_* ≤ 80 and acceptable to desirable discriminatory power *r_it_* >.3.

### 3.6 Item reduction 2 (sample 1)

To enhance usability, the scales were further reduced in sample 1 to a maximum of three items per scale. For reduction, discriminatory power, item difficulty, and Cronbach’s alpha were considered and only the best items were retained. Detailed characteristics of the reduced scales are found in **Table 6**. The final items and scoring instructions of the COPMI-SQ-r are found in **Table 7**.

**Table 6:** Results of further scale and item analyses (sample 1).

		N	M (SD) [min; max]	Item no.	α	r_it_ (M (SD) [min; max])	P_i_ (M (SD) [min; max])
Factor 1	Experienced SBA	380	24.34 (25.30) [1; 101]	3	.67	.49 (.03) [.45;.52]	23.34 (3.44) [20.40; 27.12]
Factor 2	Affiliate stigma	380	44.67 (32.03) [1; 101]	3	.82	.67 (.03) [.64;.71]	43.67 (3.93) [40.65; 48.11]
Factor 3	Shame	380	30.29 (27.60) [1; 101]	3	.75	.59 (.11) [.47;.68]	29.29 (9.38) [20.46; 39.13]
Factor 6	Anticipated SBA	380	31.24 (27.01) [1; 101]	3	.75	.59 (.15) [.42;.70]	30.24 (9.4) [22.72; 40.78]
Total***		380	32.63 (21.93) [1; 92.17]	12	.87	.56 (.07) [.38;.63]	31.63 (9.87) [20.40; 48.11]
Factor 4	Healthcare	181	72.86 (25.57) [1.33; 101]	3	.81	.66 (.02) [.64;.69]	71.86 (6.15) [64.81; 76.13]
Factor 7	Social support	380	42.86 (30.26) [1; 101]	2	.67*	.67 (.00) [.67;.67]	41.86 (3.86) [39.13; 44.59]
Total		380**	37.12 (19.68) [1; 86.93]	17	.84	.45 (.13) [.21;.61]	39.94 (17.77) [20.4; 76.13]

Item no., number of items; α, Cronbach’s alpha; *r_it_*, corrected item-whole correlation; *P_i_*, item difficulty. *Spearman Brown coefficient *r_SB_*. **Items in factor 4 N = 181. ***Without factor 4 and factor 7.

**Table 7:** COPMI-SQ-r.

Experienced stigma by association		
	Weil meine Mutter/mein Vater eine psychische Erkrankung hat, …	*Because my mother/father has a mental illness, …*
ESBA_01	… haben andere Angst vor meiner Mutter/meinem Vater oder mir.	*… others are afraid of my mother/father or me.*
ESBA_02	…sagen andere verletzende Sachen über mich oder meine Mutter/meinen Vater.	*… others say hurtful things about me or my mother/father.*
ESBA_03	Ich fühle mich als würde ich ein Schild mit mir herumtragen: “Er/Sie hat eine Mutter/einen Vater mit einer psychischen Erkrankung”	*I feel like I’m carrying around a sign: “He/she has a mother/father with a mental illness”.*

Affiliate stigma		
	Weil meine Mutter/mein Vater eine psychische Erkrankung hat, …	*Because my mother/my father has a mental illness, …*
AS_01	… fühle ich mich weniger wert.	*… I feel less worthy.*
AS_02	…denke ich, dass meine Familie nicht richtig ist.	*… I don’t think my family is normal.*
AS_03	… fühle ich mich schuldig.	*… I feel guilty.*

Shame		
SH_01 (i)	Ich habe kein Problem damit, meinen Freund*innen meine (erkrankte) Mutter/meinen (erkrankten) Vater vorzustellen.	*I have no problem introducing my (ill) mother/father to my friends.*
SH_02	Mir ist es peinlich, dass meine Mutter/mein Vater eine psychische Erkrankung hat.	*I’m embarrassed that my mother/father has a mental illness.*
SH_03	Ich schäme mich dafür, dass meine Mutter/mein Vater nicht wie andere Mütter/Väter ist.	*I’m ashamed that my mother/father isn’t like other mothers/fathers*

Anticipated stigma by association		
	Wenn andere von der Erkrankung meiner Mutter/meines Vaters erfahren würden, …	*If other people found out about my mother’s/father’s illness, …*
ASBA_01	… würden sie hinter meinem Rücken schlecht über die Erkrankung meiner Mutter/meines Vaters reden.	*… they’d speak badly about my mother’s/father’s illness behind my back*
ASBA_02	… würden sie über mich lästern.	*… they would bad-mouth me.*
ASBA_03 (i)	… würde das an ihrem Verhalten mir gegenüber nichts ändern	*… it wouldn’t change their behavior towards me.*

Healthcare (additional screener items)		
	Wenn meine Mutter/mein Vater aufgrund der psychischen Erkrankung im Krankenhaus war, …	*When my mother/father was in hospital because of their mental illness, …*
HC_01 (i)	… konnte ich das Personal immer ansprechen, wenn ich Fragen zur Erkrankung meiner Mutter/meines Vaters hatte.	*… I could always approach the staff if I had any questions about my mother’s/father’s illness.*
HC_02 (i)	… fühlte ich mich vom Krankenhauspersonal gut einbezogen und informiert.	*… I felt well integrated and informed by the hospital staff.*
HC_03 (i)	… war die Beziehung zwischen mir und dem Krankenhauspersonal gut.	*… the relationship between me and the hospital staff was good.*

Social support (additional screener items)		
SS_01 (i)	Es gibt Leute, mit denen ich über meine Ängste und Sorgen reden kann.	*There are people I can talk to about my fears and worries.*
SS_02 (i)	Wenn ich wegen der Erkrankung meiner Mutter/meines Vaters Hilfe brauche, gibt es Personen. mit denen ich sprechen kann.	*If I need help because of my mother’s/father’s illness, there are people I can turn to.*

scoring 1–101; (i) inverted item, scoring 101–1.

Factor 1 (“Experienced SBA”), factor 4 (“Healthcare”), and factor 7 (“Social support”): Since these scales already consisted of only two or three items, no further items were removed.

Factor 2 (“Affiliate stigma”): Step-by-step, eight items were removed. After removal, Cronbach’s alpha was α = .82. Discriminatory power was acceptable to (very) good (.64 ≤ *r_it_* ≤.71) and item difficulties ranged from *P_i_* = 40.65 to *P_i_* = 48.11.

Factor 3 (“Shame”): Step-by-step, three items were removed. After removal, Cronbach’s alpha was α = .75. Discriminatory power was (almost) good (.47 ≤ *r_it_* ≤.68), and item difficulties range from *P_i_* = 20.46 to *P_i_* = 39.13.

Factor 6 (“Anticipated SBA”): One item was removed. After removal, Cronbach’s alpha was α = .75. Discriminatory power was acceptable to good (.42 ≤ *r_it_* ≤.70), and item difficulties range from *P_i_* = 22.72 to *P_i_* = 40.78.

Total scale: Cronbach’s alpha was α = .84 for the total scale. Removing one item from factor 4 would have increased Cronbach’s alpha to α = .85. All items showed desirable item difficulties 20 < *P_i_* < 80. Four items showed inacceptable discriminatory power *r_it_* <.3. Since all these items loaded on factor 4 (“Healthcare”) and factor 7 (“Social support”), those factors were excluded when calculating the total scale. Due to content considerations, the subscales were nevertheless retained as separate screening scales. After excluding factors 4 and 7, Cronbach’s alpha was α = .87. Removing any additional item would not have improved Cronbach’s alpha. All remaining items showed desirable item difficulties 20 < *P_i_* < 80 and acceptable to desirable discriminatory power.38 ≤ *r_it_* ≤.63. All remaining items and their scale allocation are found in **Table 7**.

### 3.7 Results of CFA (sample 2)

Testing the theoretically derived four factor structure (model A*) with a CFA in sample 2 resulted in an inadequate model fit (χ^2^(2138) = 7,093.22, *p* <.001; RMSEA = .065 (90% CI = .063–.066); SRMR = .101; CFI = .667; AIC = 334 651.99) (see **Table 8**, model A*). All calculated models based on EFA and item analyses revealed a better model fit according to AIC than the theoretically derived four factor structure (see **Table 8**, models A*-F*). According to RMSEA (RMSEA ≤.062), SRMR (SRMR ≤.056), and CFI (CFI ≥.942), the COPMI-SQ-r with and without the additional screening scales showed a good model fit. Regarding AIC, a better model fit was observed for the COPMI-SQ-r without the additional screening scales (AIC = 60 008.05).

**Table 8:** Results of confirmatory factor analyses (sample 2).

Model	χ^2^	*df*	*p*	χ^2^/*df*	RMSEA [90% CI]	SRMR	CFI	AIC
A*	7,093.22	2,138	<.001	3.32	.065 [.063;.066]	.101	.667	334,651.99
B*	2,950.16	881	<.001	3.35	.065 [.063;.068]	.77	.768	219,428.19
C*	1,472.05	362	<.001	4.07	.075 [.071;.078]	.089	.746	145,786.94
D*	1,270.86	246	<.001	5.17	.087 [.083;.091]	.096	.723	120 996.64
E*	247.29	104	<.001	2.38	.050 [.043;.057]	.056	.942	84,798.78
F*	149.19	48	<.001	3.11	.062 [.052;.072]	.049	.945	60,008.05

A, original COPMI-SQ; B, seven-factor model derived by EFA; C, six-factor model derived by item analyses (all scales); D, four-factor model derived by item analyses (without additional screening scales); E, COPMI-SQ-r with additional screening scales; F, COPMI-SQ-r without additional screening scales. *Analyses were conducted in sample 2; *χ^2^*, model chi square; df, degrees of freedom; *p*, p-value; RMSEA, root mean square error of approximation; SRMR, standardized root mean square residual; CFI, comparative fit index; AIC, Akaike information criterion.

### 3.8 Results of Multiple-Group CFA (sample 1 and sample 2)

In the combined sample of adolescents who reported growing up with and without a parent with a mental illness (sample 1 and sample 2), configural invariance was observed (χ^2^ (208) = 481.58, p < .001; CFI = .939; RMSEA = .053 (90% CI = .047-.059); SRMR = .056), but metric invariance could not be established (Δχ^2^ (11) = 35.38; p < .001).

### 3.9 Item analyses (sample 2)

Item and scale analyses concerning their difficulty, discriminatory power, and reliability were also conducted for the revised instrument in sample 2. Detailed characteristics of the scales are found in **Table 9**. The final items and scoring instructions of the parallel version of the COPMI-SQ-r are found in **Table 10**.

**Table 9:** Results of item analyses (sample 2).

		N	M (SD) [min; max]	Item no.	α	r_it_ (M (SD) [min; max])	P_i_ (M (SD) [min; max])
Factor 1	Experienced SBA	550	60.81 (21.72) [1; 101]	3	.68	.5 (.08) [.43;.59]	59.81 (6.59) [53.62; 66.74]
Factor 2	Affiliate stigma	550	64.97 (22.28) [1; 101]	3	.78	.62 (.05) [.57;.67]	63.97 (.98) [62.88; 64.75]
Factor 3	Shame	550	62.63 (19.2) [1; 101]	3	.69	.52 (.21) [.28;.66]	61.63 (3.38) [57.78; 64.13]
Factor 6	Anticipated SBA	550	55.67 (22.2) [1; 101]	3	.64	.46 (.19) [.25;.59]	54.67 (6.29) [47.78; 60.11]
Total**		550	61.02 (16.37) [4.83; 95.33]	12	.84	.51 (.12) [.24;.61]	60.02 (5.49) [47.78; 66.74]
Factor 4	Healthcare	550	55.11 (20.01) [1; 101]	3	.75	.59 (.01) [.58;.59]	54.11 (5.34) [50.18; 60.19]
Factor 7	Social support	550	47.64 (23.12) [1; 101]	2	.59*	.61 (.00) [.61;.61]	46.64 (1.49) [45.59; 47.69]
Total		550	58.4 (13.09) [3.71; 94.41]	17	.81	.4 (.13) [.19;.55]	57.4 (6.79) [45.59; 66.74]

Item no., number of items; α, Cronbach’s alpha; *r_it_*, corrected item-whole correlation; *P_i_*, item difficulty. *Spearman Brown coefficient *r_SB_*.**Without factor 4 and factor 7.

**Table 10:** COPMI-SQ-r (parallel version).

Experienced stigma by association		
	Weil das Elternteil eines Kindes eine psychische Erkrankung hat, …	*Because the parent of a child has a mental illness, …*
ESBA_01	… haben andere Angst vor dem Elternteil oder dem Kind.	*… others are afraid of the parent or the child.*
ESBA_02	…sagen andere verletzende Sachen über das Kind oder sein Elternteil.	*… others say hurtful things about the child or the parent.*
ESBA_03	Das Kind fühlt sich, als würde es ein Schild mit sich herumtragen: “Er/Sie hat eine Mutter/einen Vater mit einer psychischen Erkrankung”	*The child feels like he’s carrying around a sign: “He/she has a mother/father with a mental illness”.*

Affiliate stigma		
	Weil das Elternteil eines Kindes eine psychische Erkrankung hat, …	*Because the parent of a child has a mental illness, …*
AS_01	… fühle das Kind sich weniger wert.	*… the child feels less worthy.*
AS_02	…denkt das Kind, dass seine Familie nicht richtig ist.	*… the child doesn’t think his family is normal.*
AS_03	… fühle das Kind sich schuldig.	*… the child feels guilty.*

Shame		
SH_01 (i)	Das Kind hat kein Problem damit, seinen Freund*innen sein (erkranktes) Elternteil vorzustellen.	*The child has no problem introducing the (ill) parent to his friends.*
SH_02	Dem Kind ist es peinlich, dass sein Elternteil eine psychische Erkrankung hat.	*The child is embarrassed that his parent has a mental illness.*
SH_03	Das Kind schämt sich dafür, dass sein Elternteil nicht wie andere Eltern ist.	*The child is ashamed that his parent isn’t like other parents.*

Anticipated stigma by association		
	Wenn andere von der psychischen Erkrankung des Elternteils eines Kindes erfahren würden, …	*If other people found out about the child’s parent’s mental illness, …*
ASBA_01	… würden sie hinter dem Rücken des Kindes schlecht über die Erkrankung seines Elternteils reden.	*… they’d speak badly about the parent’s illness behind the child’s back*
ASBA_02	… würden sie über das Kind lästern.	*… they would bad-mouth the child.*
ASBA_03 (i)	… würde das an ihrem Verhalten dem Kind gegenüber nichts ändern	*… it wouldn’t change their behavior towards the child.*

Healthcare (additional screener items)		
	Wenn das Elternteil eines Kindes aufgrund einer psychischen Erkrankung im Krankenhaus war, …	*When the parent of a child was in hospital because of their mental illness, …*
HC_01 (i)	… konnte das Kind das Personal immer ansprechen, wenn es Fragen zur Erkrankung seines Elternteils hatte.	*… the child could always approach the staff if it had any questions about his parent’s illness.*
HC_02 (i)	… fühlte das Kind sich vom Krankenhauspersonal gut einbezogen und informiert.	*… the child felt well integrated and informed by the hospital staff.*
HC_03 (i)	… war die Beziehung zwischen dem Kind und dem Krankenhauspersonal gut.	*… the relationship between the child and the hospital staff was good.*

Social support (additional screener items)		
SS_01 (i)	Es gibt Leute, mit denen das Kind über seine Ängste und Sorgen reden kann.	*There are people the child can talk to about his fears and worries.*
SS_02 (i)	Wenn das Kind wegen der Erkrankung seines Elternteils Hilfe braucht, gibt es Personen. mit denen es sprechen kann.	*If the child needs help because of his parent’s illness, there are people the child can turn to.*

scoring 1–101; (i) inverted item, scoring 101–1.

Factor 1 (“Experienced SBA”): Cronbach’s alpha was almost acceptable with α = .68. Discriminatory power was acceptable to good (.43 ≤ *r_it_* ≤.59), and item difficulties ranged from *P_i_* = 53.62 to *P_i_* = 66.74.

Factor 2 (“Affiliate stigma”): Cronbach’s alpha was acceptable with α = .78. Discriminatory power was good (.57 ≤ *r_it_* ≤.67), and item difficulties ranged from *P_i_* = 62.88 to *P_i_* = 64.75.

Factor 3 (“Shame”): Cronbach’s alpha was almost acceptable with α = .69. Discriminatory power was almost acceptable to good (.28 ≤ *r_it_* ≤.66) and item difficulties ranged from *P_i_* = 57.78 to *P_i_* = 64.13.

Factor 6 (“Anticipated SBA”): Cronbach’s alpha was almost acceptable with α = .64. Discriminatory power was almost acceptable to good (.25 ≤ *r_it_* ≤.59), and item difficulties ranged from *P_i_* = 47.78 to *P_i_* = 60.11.

Factor 4 (“Healthcare”): Cronbach’s alpha was acceptable with α = .75. Discriminatory power was almost good (.58 ≤ *r_it_* ≤.59), and item difficulties ranged from *P_i_* = 50.18 to *P_i_* = 60.19.

Factor 7 (“Social support”): Since factor 7 consists of only two items, Spearman’s brown coefficient (*r_SB_*) was calculated as reliability coefficient. Spearman’s brown coefficient was acceptable with *r_SB_* = .59. The items showed desirable discriminatory power (*r_it_* = .61), and item difficulties ranged from *P_i_* = 45.59 to *P_i_* = 47.69.

Total scale: Cronbach’s alpha was α = .81 for the total scale. Item difficulties ranged from *P_i_* = 45.59 to *P_i_* = 66.74. Five items showed inacceptable discriminatory power *r_it_* <.3. All these items loaded on factor 4 (“Healthcare”) and factor 7 (“Social support”). After excluding these factors from calculating the total scale, Cronbach’s alpha was α = .84. Item difficulties ranged from *P_i_* = 47.78 to *P_i_* = 66.74. Discriminatory power was acceptable to good (.32 ≤ *r_it_* ≤.61) for all but one item (*r_it_* = .24).

## 4 Discussion

Mental disorders are still frequently stigmatized in society, which has negative consequences not only for the people with a mental disorder themselves but also for their family members. This is known as *stigma by association* (SBA) (9, 13–17). Children of parents with a mental illness carry an increased risk to experience SBA, one of the assumed mechanisms of the transgenerational transmission of mental disorders (25–29). To our knowledge, the COPMI-SQ is the first questionnaire to have been developed assessing such stigma-specific experiences of adolescents who grow up with a parent with a mental illness (30). Promising psychometric properties were reported in an initial small pilot study (30). In the current paper, we investigated the factorial structure and psychometric properties of this instrument in a larger sample and present a shortened and revised version—the COPMI-SQ-r. The structure of the COPMI-SQ-r was identified in a sample of adolescents who reported growing up with a parent with a mental illness (sample 1): Using CFA, the structure was replicated in an independent sample of adolescents who reported not growing up with a parent with a mental illness (sample 2). Furthermore, configural invariance was established in a multiple-group CFA in the combined sample of adolescents who reported growing up with and without a parent with a mental illness (sample 1 and sample 2). In the sample of adolescents who reported growing up with a parent with a mental illness (sample 1), psychometric properties of the COPMI-SQ-r were found to be acceptable to good. In the sample of adolescents who reported not growing up with a parent with a mental illness (sample 2), psychometric properties of the COPMI-SQ-r were found to be almost acceptable to good.

The final version of the COPMI-SQ-r consists of 12 items that load on the four main subscales “Experienced SBA,” “Affiliate stigma,” “Shame,” and “Anticipated SBA” (three items each). *Experienced stigma*, *anticipated stigma*, and *internalized stigma* have been identified as the most relevant mechanisms applying to the primary recipients of stigmatization (5), and it has been argued that SBA involves the same mechanisms (32).

The subscale “Experienced SBA” measures personally experienced stereotypes, prejudices, and discrimination in the past or present. *Having unmet emotional needs* (e.g., inappropriate language and contents, withdrawal and rejection) and *experiencing hostile behaviors of others* were identified as important aspects of “Experienced SBA” (32) that are both represented in the Experienced SBA subscale. Furthermore, one item of this subscale relates to the *belief of being contaminated*, which was originally thought to be part of the “Affiliate stigma” dimension (30). In the literature, children of parents with a mental illness have frequently been described as experiencing “contamination stigma” (20, 22, 32). On the one hand, contamination can refer to a “fear of inheriting their parent’s illness” (55)—an aspect that, due to item reduction, is no longer covered in the COPMI-SQ-r. On the other hand, contamination can also refer to the fear adolescents experience as closely connected to the parent with a mental illness and thus, also be considered to be “crazy” (56). Since the present item (“I feel like I’m carrying around a sign: ‘He/she has a mother/father with a mental illness.’”) refers not to a fear but to actual experiences of adolescents being perceived differently by others because of their parent’s mental illness, it makes sense that this item is incorporated within the subscale “Experienced SBA”.

The subscale “Anticipated SBA” captures the expectation of stigmatization in the future, and *fearing hostile behaviors of others*, *fear of negative attitudes and ascriptions*, and *fearing others’ lack of understanding and rejections* were identified as important aspects of “Anticipated SBA” in adolescents who grow up with a parent with a mental illness (32). Even though the present items were originally devised to specifically assess the aspect of *fearing hostile behaviors* (30), we find that they adequately capture “Anticipated SBA” in general. Concerning stigmatization, discrimination (e.g., hostile behaviors) is seen as the behavioral reaction to stereotypes and prejudices (5, 6). Attitudes are considered to be an individual’s positive or negative evaluation of a psychological object (57), which largely resembles the definition of prejudices as the approval of an existing stereotype in association with negative emotions (5, 6). The influence of attitudes on behavior has been well established in the theory of planned behavior (58). Therefore, we consider hostile behavior to be both the observable and anticipatable consequence of negative attitudes and of other’s lack of understanding.

“Affiliate stigma” can be understood as the SBA version of *internalized stigma* (33), and refers to the internalization of stereotypes and prejudices to the self (32). *Perceiving themselves as being contaminated* and *perceiving themselves as being inferior* have been identified as important aspects of “Affiliate Stigma” in adolescents who grow up with a parent with a mental illness (32). Stigmatization is understood as a process in which individuals are assigned to a group on the basis of a discrediting characteristic or trait and are subsequently devalued (3, 4). For adolescents who grow up with a parent with a mental illness, this seems to lead to a feeling of being less valuable and abnormal (32), a feeling the present subscale captures.

“Shame” was identified as a separate subscale of the COPMI-SQ-r in this paper. Most of the items in this subscale were originally part of the COPMI-SQ’s “Affiliate stigma” subscale. A recent integrative review identified that stigma experiences of children and adolescents who grow up with a parent with a mental illness often result in feelings of shame and embarrassment, and subsequently the attempt to hide the parental mental illness (21). This implies that “Shame” is more of a consequence of the other stigma dimensions, and therefore, it makes sense to assess it in a separate subscale.

Furthermore, the COPMI-SQ-r includes two additional screening scales: “Healthcare” (three items) and “Social support” (two items). The “Healthcare” subscale consists of the only items retained from the COPMI-SQ’s “Structural discrimination” subscale. Structural discrimination refers to the perpetuation of stigmatization through societal and policy structures (34). The original “Structural discrimination” subscale included a heterogeneous item pool (e.g., concerning media, school, and healthcare) and was not necessarily specific to the stigma experiences of adolescents who grow up with a parent with a mental illness (“Mental illness is portrayed negatively in the media.”). Due to their unspecificity and high heterogeneity, most of the items were already eliminated during the EFAs in sample 1 as they did not load on specific factors. Considering that stigmatization is a major issue in healthcare and has negative effects on treatment seeking and recovery (59), we nevertheless decided to keep these remaining three items as a separate screening scale to assess specific stigmatization experiences of adolescents who grow up with a parent with a mental illness in the healthcare sector. In line with this, two items were found to load on a specific “Social support” factor. Since social support is known to be an important protective factor for mental health (60, 61), we also decided to keep these items as a separate screening scale.

### 4.1 Limitations

Several limitations need to be considered regarding the present study. Having reduced the COPMI-SQ’s item numbers from initially 109 to now 17 (including the five screener items), some aspects of SBA concerning children and adolescents who grow up with a parent with a mental illness that appeared to be important in the systematic review (32) (e.g., fear of contamination by the parental mental disorder, structural discrimination in school or media) are no longer assessable with the COPMI-SQ-r. Nevertheless, the COPMI-SQ-r still captures the most relevant different aspects of stigma by association as reported in the literature (5, 20, 21, 32). Furthermore, the item reduction enhances its usability for both research and clinical practice. Recruiting adolescents who grow up with a parent with a mental illness into research studies is challenging (35, 36), but by social media advertising, we were able to recruit a large sample of adolescents who reported growing up with at least one parent with a mental illness. While our sample is large, it was not large enough to randomly divide it into two subsample to explore and test the factorial structure. Thus, an independent sample of adolescents who reported not growing up with a parent with a mental illness and who answered a parallel version of the COPMI-SQ that only differed on item stem was used to validate the derived factorial structure. In a multiple-group CFA, configural invariance was observed, but metric invariance could not be established. This implies that even though the factorial structure is the same between the different groups, results from using the COPMI-SQ-r in samples of adolescents who report growing up with and without a parent with a mental illness should not be compared (62, 63). In future studies, the factorial structure of COPMI-SQ-r should also be tested in other independent samples with adolescent who report growing up with a parent with a mental illness. Furthermore, our sample is self-selected and representativeness cannot be assumed (35). Furthermore, all data were collected only through self-report from the participating adolescents. Thus, reported parental symptoms and diagnoses have not been clinically validated. In addition, over 90% of participating adolescents indicated in sample 1 having a mental illness themselves. This number is much higher than reported in other studies (24, 27, 30), but again, one must be bear in mind that those are not clinically validated diagnoses and that the sample might be biased due to the recruitment strategy used (35). On the one hand, this high number of young people already affected emphasizes the need for preventive interventions to break the vicious circle of transgenerational transmission at an early stage (29, 64, 65). On the other hand, this also means that adolescents’ responses may be cofounded by stigma experiences regarding their own mental disorder. Future studies should therefore aim to include more representative samples with clinically validated diagnoses and with young people who do not experience mental health challenges themselves. Nevertheless, the reported rate of adolescents (59.4%), mothers (51.6%), and fathers (30.8%) in sample 1 being treated for their mental disorders indicates that the reported problems and symptoms are for the most part clinically relevant. As the COPMI-SQ-r has been only validated in German so far, its results are not generalizable to other cultures. In future studies, the existing English translation should therefore be used as a starting point to validate the COPMI-SQ-r in other languages and cultures. Most of the COPMI-SQ-r items reveal high item difficulty, meaning that few participants approved very highly of the items. To assess a broader range of stigmatization experiences of adolescents who grow up with a parent with a mental illness, future revisions of the instrument should ideally include items with lower item difficulty. Finally, yet importantly, as research in this field progresses, the COPMI-SQ-r’s concurrent and discriminant validity should be investigated as well as its retest reliability. Only factorial and content validity of the instrument has been ensured so far through the theory-based approach that was complemented by an expert panel during the piloting phase (30).

### 4.2 Implications

To reduce the stigma of mental illness in general and SBA in children and adolescents affected by a parental mental illness specifically, general anti-stigma campaigns and targeted interventions to reduce SBA in affected children and adolescents are needed (1, 31). One example is the Australian StigmaBeat project (66). In a participatory approach with young people who are affected by parental mental health challenges, films aiming at reducing mental health stigma were developed. The COPMI-SQ-r could be used to help develop, adapt, and evaluate activities such as StigmaBeat.

### 4.3 Conclusion

We have described here the development and validation of the COPMI-SQ-r transparently and in detail. The COPMI-SQ-r is a theoretically grounded, methodologically sound, reliable, and economic questionnaire to assess SBA in adolescents who grow up with a parent with a mental illness, which can be applied in both research and clinical practice, and it will enable us to better understand the specific stigma experiences of adolescents who grow up with a parent with a mental illness.

## Data availability statement

The raw data supporting the conclusions of this article will be made available by the authors, without undue reservation.

## Ethics statement

The studies involving humans were approved by Ethics Committee of the Department of Psychology at Philipps University Marburg. The studies were conducted in accordance with the local legislation and institutional requirements. Written informed consent for participation in this study was provided by the participants’ legal guardians/next of kin.

## Author contributions

MS: Conceptualization, Data curation, Formal analysis, Methodology, Project administration, Writing – original draft, Writing – review & editing. L-MD: Conceptualization, Writing – review & editing. HC: Conceptualization, Writing – review & editing, Supervision.

## Supplementary material

SUPPLEMENTARY TABLE 1 COPMI-SQ reduction steps.
