# Supplementary material for: Children of parents with a mental illness – stigma questionnaire: validation and revision
Source: Front Psychiatry. 2024 Jun 17;15:1376627. doi: 10.3389/fpsyt.2024.1376627 (PMC11215172; doi:10.3389/fpsyt.2024.1376627)
Supplement: SUPPLEMENTARY TABLE 1 — COPMI-SQ reduction steps. [file Table_1.docx]

**Experienced SBA**

|  | Original item | Translation | Scoring | Retention / Removal | Comment | COPMI-SQ r |
| --- | --- | --- | --- | --- | --- | --- |
|  | *Preceded by:*  Weil meine Mutter/mein Vater eine psychische Erkrankung hat, … | *Preceded by:*  Because my mother/father has a mental illness, … |  |  |  |  |
| ESBA_01 | …machen sich andere über meine Mutter/meinen Vater lustig. | …others make fun of my mother/father. | 1-101 | Removal | Item Reduction 1 Factor 1  Item difficulty P_i_ < 20 |  |
| ESBA_02 | …reden andere hinter meinem Rücken über die Erkrankung meiner Mutter/meines Vaters. | …others talk about my mother's/father's illness behind my back. | 1-101 | Removal | Item Reduction 1 Factor 1  Item difficulty P_i_ < 20 |  |
| ESBA_03 | …lästern andere über mich. | …others say awful things about me. | 1-101 | Removal | Item Reduction 1 Factor 1  Item difficulty P_i_ < 20 |  |
| ESBA_04 | …lachen andere mich aus. | …others laugh at me. | 1-101 | Removal | Item Reduction 1 Factor 1  Item difficulty P_i_ < 20 |  |
| ESBA_05 | …tratschen andere das weiter. | …others gossip about it. | 1-101 | Removal | Item Reduction 1 Factor 1  Item difficulty P_i_ < 20 |  |
| ESBA_06 | …gehen andere mir aus dem Weg. | …others avoid me. | 1-101 | Removal | Item Reduction 1 Factor 1  Item difficulty P_i_ < 20 |  |
| ESBA_07 | …haben andere Angst vor meiner Mutter/meinem Vater oder mir. | …others are afraid of my mother/father or me. | 1-101 | Retention |  | Experienced SBA |
| ESBA_08 | …möchten meine Freund*innen nicht mehr mit mir befreundet sein. | …my friends no longer want to be friends with me. | 1-101 | Removal | Item Reduction 1 Factor 1  Item difficulty P_i_ < 20 |  |
| ESBA_09 | …wollen sich meine Mitschüler*innen/Kommiliton*innen/ Arbeitskolleg*innen nicht mehr mit mir treffen. | …my classmates/colleagues/ work colleagues no longer want to get together with me. | 1-101 | Removal | Item Reduction 1 Factor 1  Item difficulty P_i_ < 20 |  |
| ESBA_10 | …ärgern mich meine Mitschüler*innen/Kommiliton*innen/ Arbeitskolleg*innen. | …my classmates/colleagues/ work colleagues aggravate me. | 1-101 | Removal | Item Reduction 1 Factor 1  Item difficulty P_i_ < 20 |  |
| ESBA_11 | …werde ich in der Schule/Uni/auf der Arbeit gemobbt. | …I’m bullied at school/university/ work. | 1-101 | Removal | EFA 3  Crossloadings with difference ≤ .2 |  |
| ESBA_12 | …wissen andere nicht, wie sie passend darauf reagieren/damit umgehen sollten. | …others do not know how to react to or deal with it appropriately. | 1-101 | Removal | EFA 4  All factor loadings < .3 |  |
| ESBA_13 | …raten andere mir, selbst keine Kinder zu bekommen. | …others advise me not to have children myself. | 1-101 | Removal | EFA 1  All factor loadings < .3 |  |
| ESBA_14 | …sagen andere verletzende Sachen über mich oder meine Mutter/meinen Vater. | …others say hurtful things about me or my mother/father. | 1-101 | Retention |  | Experienced SBA |
| ESBA_15 | Es gibt Leute, mit denen ich über meine Ängste und Sorgen reden kann. | There are people I can talk to about my fears and worries. | 101-1 | Additional screening scales |  | Social support |
| ESBA_16 | Andere Leute möchten **nicht** mit mir über die Erkrankung meiner Mutter/meines Vaters sprechen. | Other people don't want to talk to me about my mother's/father's illness. | 1-101 | Removal | EFA 1  All factor loadings < .3 |  |
| ESBA_17 | Wenn ich wegen der Erkrankung meiner Mutter/meines Vaters Hilfe brauche, gibt es Personen mit denen ich sprechen kann. | If I need help because of my mother's/father's illness, there are people I can turn to. | 101-1 | Additional screening scales |  | Social support |

**Anticipated SBA**

|  | Original item | translation | scoring | Retention / Removal | comment | COPMI-SQ r |
| --- | --- | --- | --- | --- | --- | --- |
|  | *Preceded by:*  Wenn andere von der Erkrankung meiner Mutter/meines Vaters erfahren würden, … | *Preceded by:*  If others found out about my mother's/father's illness, … |  |  |  |  |
| ASBA_01 | …würden sie sich über meine Mutter/meinen Vater lustig machen. | …they’d make fun of my mother/father. | 1-101 | Removal | EFA 3  Crossloadings with difference ≤ .2 |  |
| ASBA_02 | …würden sie hinter meinem Rücken schlecht über die Erkrankung meiner Mutter/meines Vaters reden. | …they’d speak badly about my mother's/father's illness behind my back | 1-101 | Retention |  | Anticipated SBA |
| ASBA_03 | …würden sie über mich lästern. | …they would bad-mouth me. | 1-101 | Retention |  | Anticipated SBA |
| ASBA_04 | …würde das an ihrem Verhalten mir gegenüber **nichts** ändern | …it wouldn’t change their behavior towards me. | 101-1 | Retention |  | Anticipated SBA |
| ASBA_05 | …würden sie mich auslachen. | …they’d laugh at me. | 1-101 | Removal | Item Reduction 1 Factor 5  Item difficulty P_i_ < 20 |  |
| ASBA_06 | …würden sie das für sich behalten. | …they’d keep it to themselves. | 101-1 | Removal | Item Reduction 2  Factor 6  Step 1 |  |
| ASBA_07 | …würden sie mir aus dem Weg gehen. | …they‘d avoid me. | 1-101 | Removal | EFA 3  Crossloadings with difference ≤ .2 |  |
| ASBA_08 | …würden sie Angst vor meiner Mutter/meinem Vater oder mir bekommen | …they’d become afraid of my mother/father or me. | 1-101 | Removal | Item Reduction 2  Factor 3  Step 3 |  |
| ASBA_09 | …würden sich meine Mitschüler*innen/Kommiliton*innen/ Arbeitskolleg*innen nicht mehr mit mir treffen wollen. | …my classmates/fellow students/ colleagues would no longer want to get together with me. | 1-101 | Removal | Item Reduction 1 Factor 5  Item difficulty P_i_ < 20 |  |
| ASBA_10 | …würden mich meine Mitschüler*innen/Kommiliton*innen/ Arbeitskolleg*innen ärgern. | …my classmates/fellow students/ colleagues at work would get angry with me. | 1-101 | Removal | Item Reduction 1 Factor 5  Item difficulty P_i_ < 20 |  |
| ASBA_11 | …würde ich in der Schule/Uni/auf der Arbeit gemobbt werden. | …I’d be bullied at school/university/work. | 1-101 | Removal | Item Reduction 1 Factor 5  Item difficulty P_i_ < 20 |  |
| ASBA_12 | …würden sie mir raten, selbst keine Kinder zu bekommen. | …others would advise me not to have children myself. | 1-101 | Removal | EFA 5  Crossloadings with difference ≤ .2 |  |
| ASBA_13 | …würden sie verletzende Sachen über mich oder meine Mutter/meinen Vater sagen. | …they’d say hurtful things about me or my mother/father. | 1-101 | Removal | EFA 3  Crossloadings with difference ≤ .2 |  |
| ASBA_14 | Wenn Fachleute (Jugendamt/Psycholog*innen/ Sozialarbeiter*innen. etc.) von der Erkrankung meiner Mutter/meines Vaters erfahren würden,  könnte ich weiterhin zu Hause wohnen bleiben. | If professionals (youth welfare office/psychologists/social workers. etc.) found out about my mother's/father's illness, I could still keep living at home. | 101-1 | Removal | EFA 4  All factor loadings < .3 |  |
| ASBA_15 | Ich bringe selten neue Freund*innen mit nach Hause, aus Angst, dass sie nicht mehr mit mir befreundet sein wollen, wenn sie von der Erkrankung meiner Mutter/meines Vaters erfahren. | I rarely bring new friends home for fear that they won't want to be friends with me anymore. | 1-101 | Removal | EFA 3  Crossloadings with difference ≤ .2 |  |
| ASBA_16 | Ich habe kein Problem damit, meinen Freund*innen meine (erkrankte) Mutter/meinen (erkrankten) Vater vorzustellen. | I have no problem introducing my (ill) mother/father to my friends. | 101-1 | Retention |  | Shame |

**Affiliate Stigma**

|  | Original item | translation | scoring | Retention / Removal | comment | COPMI-SQ r |
| --- | --- | --- | --- | --- | --- | --- |
|  | *Preceded by:*  Weil meine Mutter/mein Vater eine psychische Erkrankung hat, … | *Preceded by:*  Because my mother/father has a mental illness, … |  |  |  |  |
| AS_01 | …denke ich, mit mir stimmt etwas nicht. | …I think there’s something wrong with me. | 1-101 | Removal | Item Reduction 2  Factor 2  Step 8 |  |
| AS_02 | …versuche ich, mich besonders normal und unauffällig zu verhalten, damit andere mir nicht anmerken, dass ich nicht normal bin. | …I try to act particularly normal and inconspicuous so that others do not notice that I am not normal. | 1-101 | Removal | Item Reduction 2  Factor 2  Step 7 |  |
| AS_03 | …nehmen andere wahr, dass ich anders/komisch bin. | …others notice that I am different/weird. | 1-101 | Removal | Item Reduction 2  Factor 2  Step 5 |  |
| AS_04 | …habe ich Angst, mich anstecken zu können. | …I am afraid of catching the illness. | 1-101 | Removal | EFA 1  All factor loadings < .3 |  |
| AS_05 | …denke ich bei kleinsten Anzeichen, dass ich dieselbe Erkrankung habe wie meine Mutter/mein Vater. | …I think at the slightest sign that I have the same illness as my mother/father. | 1-101 | Removal | Item Reduction 2  Factor 2  Step 2 |  |
| AS_06 | …denke ich, dass ich später keine eigenen Kinder bekommen sollte. | …I don’t think I should have children of my own later on. | 1-101 | Removal | Item Reduction 2  Factor 2  Step 6 |  |
| AS_07 | …fühle ich mich weniger wert. | …I feel less worthy. | 1-101 | Retention |  | Affiliate SBA |
| AS_08 | …denke ich, dass meine Familie nicht richtig ist. | …don’t think my family is normal. | 1-101 | Retention |  | Affiliate SBA |
| AS_09 | …fühle ich mich schuldig. | …I feel guilty. | 1-101 | Retention |  | Affiliate SBA |
| AS_10 | …schäme ich mich. | …I feel ashamed. | 1-101 | Removal | EFA 5  Crossloadings with difference ≤ .2 |  |
| AS_11 | Ich fühle mich als würde ich ein Schild mit mir herumtragen: „Er/Sie hat eine Mutter/einen Vater mit einer psychischen Erkrankung“ | I feel like I'm carrying around a sign: "He/she has a mother/father with a mental illness". | 1-101 | Retention |  | Experienced SBA |
| AS_12 | Ich bin ein ganz normales Kind wie jedes andere auch. | I’m just a normal kid like any other. | 101-1 | Removal | Item Reduction 2  Factor 2  Step 1 |  |
| AS_13 | Weil ich so ein schwieriges Kind bin, ist meine Mutter/mein Vater erkrankt. | Because I am such a difficult child, my mother/father has become ill. | 1-101 | Removal | Item Reduction 2  Factor 2  Step 3 |  |
| AS_14 | Ich bin (mit-)verantwortlich dafür, dass sich der Zustand meiner Mutter/meines Vaters nicht verbessert. | I am (co-)responsible for the fact that the condition of my mother/father isn’t improving. | 1-101 | Removal | Item Reduction 2  Factor 2  Step 4 |  |
| AS_15 | Ich muss die Erkrankung meiner Mutter/meines Vaters geheim halten. | I have to keep my mother's/father's illness a secret. | 1-101 | Removal | Item Reduction 2  Factor 3  Step 1 |  |
| AS_16 | Wenn ich die Erkrankung meiner Mutter/meines Vaters beschreibe, spiele ich die Schwere der Erkrankung herunter. | When I describe my mother's/father's illness. I downplay the severity of it. | 1-101 | Removal | Item Reduction 2  Factor 3  Step 2 |  |
| AS_17 | Mir ist es peinlich, dass meine Mutter/mein Vater eine psychische Erkrankung hat. | I’m embarrassed that my mother/father has a mental illness. | 1-101 | Retention |  | Shame |
| AS_18 | Ich schäme mich dafür, dass meine Mutter/mein Vater nicht wie andere Mütter / Väter ist. | I’m ashamed that my mother/father isn’t like other mothers/fathers. | 1-101 | Retention |  | Shame |
| AS_19 | Wenn meine Mutter/mein Vater wegen ihrer/seiner Erkrankung verurteilt wird, fühle ich mich auch verurteilt. | If my mother/father is judged because of her/his illness, I feel judged too. | 1-101 | Removal | EFA 1  All factor loadings < .3 |  |

**Structural Discrimination**

|  | Original item | translation | scoring | Retention / Removal | comment | COPMI-SQ r |
| --- | --- | --- | --- | --- | --- | --- |
|  | *Preceded by:*  Wenn meine Mutter/mein Vater aufgrund der psychischen Erkrankung im  Krankenhaus war, … | *Preceded by:*  When my mother/father was in hospital because of the mental illness,… |  |  |  |  |
| STD_01 | …konnte ich das Personal immer ansprechen, wenn ich Fragen zur Erkrankung meiner Mutter/meines Vaters hatte. | …I could always approach the staff if I had any questions about my mother's/father's illness. | 101-1 | Additional screening scales |  | Health care |
| STD_02 | …hätte ich gerne mehr Informationen vom Krankenhauspersonal bekommen. | …I’d have liked to get more information from the hospital staff. | 1-101 | Removal | EFA 1  All factor loadings < .3 |  |
| STD_03 | …fühlte ich mich vom Krankenhauspersonal gut einbezogen und informiert. | …I felt well integrated and informed by the hospital staff. | 101-1 | Additional screening scales |  | Health care |
| STD_04 | …fühlte ich mich, als wäre ich dort unerwünscht. | …I felt like I was unwanted there. | 1-101 | Removal | Item Reduction 1 Factor 4  Removing item increased Cronbach’s alpha of subscale |  |
| STD_05 | …war die Beziehung zwischen mir und dem Krankenhauspersonal gut. | …the relationship between me and the hospital staff was good. | 101-1 | Additional screening scales |  | Health care |
| STD_06 | Ich denke, meiner Mutter/meinem Vater wurde durch das Gesundheitssystem nicht genug geholfen. | My mother/father wasn’t helped enough by the health system. | 1-101 | Removal | EFA 1  All factor loadings < .3 |  |

|  | *Preceded by:*  In der Schule … | *Preceded by:*  At school … |  |  |  |  |
| --- | --- | --- | --- | --- | --- | --- |
| STD_07 | …würde ich gerne mehr über psychische Erkrankungen erfahren. | …I’d like to learn more about mental illness. | 1-101 | Removal | EFA 1  All factor loadings < .3 |  |
| STD_08 | …kann ich mit meinen Lehrer*innen über die psychische Erkrankung meiner Mutter/meines Vaters sprechen. | …I can talk to my teachers about my mother's or father's illness. | 1-101 | Removal | EFA 1  All factor loadings < .3 |  |
| STD_09 | …gehen die Lehrer*innen auf mich und meine Schwierigkeiten zu Hause ein. | …the teachers respond to me and my difficulties at home. | 101-1 | Removal | EFA 1  All factor loadings < .3 |  |
| STD_10 | …fühle ich mich wegen der psychischen Erkrankung meiner Mutter/meines Vaters **nicht** benachteiligt. | …I don’t feel disadvantaged because of my mother's/father's illness. | 101-1 | Removal | EFA 1  All factor loadings < .3 |  |
|  | *Preceded by:*  In den Medien … | *Preceded by:*  In the media, … |  |  |  |  |
| STD_11 | …werden psychische Erkrankungen angemessen dargestellt. | …mental illness is portrayed appropriately. | 101-1 | Removal | EFA 1  All factor loadings < .3 |  |
| STD_12 | …werden psychische Erkrankungen negativ dargestellt. | …mental illness is portrayed negatively. | 1-101 | Removal | Item Reduction 1 Factor 6  Removing item increased Cronbach’s alpha of subscale |  |
| STD_13 | Ich erhalte von niemandem ausreichend Informationen über die psychische Erkrankung meiner Mutter/meines Vaters. | I don’t get enough information from anyone about my mother's/father's illness. | 1-101 | Removal | EFA 1  All factor loadings < .3 |  |
| STD_14 | Ich weiß genau, an welche (professionellen) Stellen ich mich wenden kann, wenn ich Hilfe wegen der Erkrankung meiner Mutter meines Vaters benötige. | I know exactly which (professional) places I can turn to if I need help because of my mother's/father's illness.… | 101-1 | Removal | EFA 2  All factor loadings < .3 |  |
| STD_15 | Es gibt ausreichend Hilfsangebote für meine Eltern und mich. | There’s enough help available for my parents and me. | 101-1 | Removal | EFA 1  All factor loadings < .3 |  |
